# Assessment of postoperative pain following single versus multiple-visit root canal treatment: A randomised clinical trial

**DOI:** 10.6026/973206300221326

**Published:** 2026-03-31

**Authors:** Monika Bhardwaj, Bhavana Koushik, Sneha Khekade, Prashanth Shetty, Sazid Hussain, Niharika Gahlod

**Affiliations:** 1 Department of Dentistry, American International Institute of Medical Sciences, Udaipur, Rajasthan, India; 2 Department of Conservative and Endodontics, Srinivas Institute of Dental Sciences, Mangalore, Karnataka, India; 3 Department of Dental, Sudha Medical College & Hospital, Jagpura, Kota, Rajasthan, India; 4 Department of Pediatric and Preventive Dentistry, Swargiya Dadasaheb Kalmegh Smruti Dental College and Hospital, Nagpur, Maharashtra, India

**Keywords:** Postoperative pain, root canal treatment, single-visit endodontics, multiple-visit treatment, irreversible pulpitis, randomised clinical trial

## Abstract

Postoperative pain following root canal procedures remains a significant clinical challenge, with on-going controversy regarding optimal single- versus multiple-visit treatment regimens for mandibular molars with symptomatic irreversible pulpitis. This study compared postoperative pain, flare-ups and analgesic consumption between single-visit (n=60) and multiple-visit (n=60) root canal therapies using standardized rotary instrumentation and warm vertical obturation in 120 matched patients. Visual Analog Scale (VAS) measurements revealed similar pain trends across both groups, peaking at 6-12 hours postoperatively with no significant differences in pain scores at any interval. Both approaches showed comparable rates of flare-ups, swelling and analgesic intake, demonstrating equivalent safety profiles and clinical outcomes. These findings advance endodontic practice by validating single-visit root canal therapy as a time-efficient alternative that maintains comparable postoperative pain control and complication rates to multiple-visit protocols.

## Background:

Root canal therapy is one of the most frequently practised cases in dentistry practice, which is meant to eradicate pulpal infection to prevent the development of periapical pathosis [1]. Nevertheless, despite major success in endodontic procedures and instrumentation, as well as obturation materials, the subject of postoperative pain after root canal treatment has been a common clinical issue that negatively impacts the quality of life of the patient and their perception of the treatment [2]. Research shows that one-fourth to half of patients report a certain level of postoperative pain and the number of those in extreme pain and in need of emergency care is 1.4-16% [3]. Aetiology of postoperative endodontic pain is multifactorial, which includes mechanical, chemical and microbial factors. The extrusion of irrigants or filling material, instrumentation of areas outside the apical foramen and retention of microorganisms in the root canal system is also contributory factors to the inflammation of the periapical tissue and resultant pain [4]. The knowledge of such processes is critical in the development of treatment regimens that would reduce the discomfort of patients and still achieve optimal clinical results. The debate on the number of appointments necessary to achieve successful root canal treatment has a long history in endodontic literature. Multiple visit treatment was traditionally promoted, especially when the periapical pathology of the teeth was present, where interappointment medication and the observation of the canal sterility before obturation could be done [5]. Advocates believe that calcium hydroxide dressing has an antimicrobial effect, lessens bacterial burden and establishes conducive circumstances to periapical healing [6]. On the other hand, the use of single-visit root canal treatment has become quite popular, backed by the fact that similar clinical results are achieved along with the possible benefits such as a shorter period of treatment, less inconvenience to the patient and the absence of risks of contamination between appointments [7]. The one-visit procedures are also consistent with the latest patient demands towards efficient and time-effective dental treatments, which are especially applicable in the current healthcare delivery models, which focus on resource optimisation [8]. Several systematic reviews and meta-analyses explored this controversy with mixed results and did not find any significant difference in the outcome of healing between single and multiple- visit therapies [9], whereas some studies found similar success rates with a minor increase in the rates of flare-ups in single-visit therapies [10]. Nevertheless, the heterogeneity of methodologies of the included studies, the definitions of success, as well as the follow-up has restricted the conclusiveness of the given analyses.

Recent studies have concentrated on particular clinical situations in which one of the two approaches can prove to be better and compared the results of the treatment of teeth having apical periodontitis because there were no significant differences in the healing rates of the teeth under different visit protocols [11]. Likewise, another review noted that evidence was not enough to identify the benefits of either methodology in the case of postoperative pain and complications [12]. Despite numerous studies, there are still a lot of gaps in the knowledge about factors that affect postoperative pain after various visit schemes. The majority of the studies analysed nonhomogeneous tooth populations with different pulpal and periapical diagnoses, which may have confounded the findings. Also, the methods of assessment of pain and when to assess pain have not been uniform in all studies [13]. Moreover, the impact of modern instrumentation and obturation methods on the postoperative pain outcomes should be evaluated constantly because technologies keep advancing. Postoperative pain is not only important to the comfort of patients. Bad pain can result in an emergency appointment, use of more healthcare and adverse effects on patient adherence to further dental care [14]. Thus, finding treatment regimens that reduce post-surgery pain and preserve treatment effectiveness is a significant clinical goal. Therefore, it is of interest to compare the intensity of postoperative pain, occurrence and duration of postoperative pain after single-visit and multiple-visit root canal treatment in mandibular molars that presented with symptomatic irreversible pulpitis. Secondary goals were to assess the rate of flare-ups, analgesic use and the presence of swelling among the treatment groups.

## Materials and Methods:

## Study design and ethical considerations:

This prospective, parallel-group, randomised clinical trial was conducted at the Department of Endodontics.

## Sample size calculation:

Sample size was determined using G*Power software (version 3.1.9.7) based on previous studies reporting mean VAS differences of 8-12 mm between treatment protocols. Assuming a clinically meaningful difference of 10 mm in VAS scores, a standard deviation of 20 mm, an alpha level of 0.05 and a power of 80%, a minimum of 52 patients per group was required. Accounting for 15% anticipated dropout rate, 60 patients were recruited per group, yielding a total sample of 120 participants.

## Participant selection:

Patients presenting to the endodontic clinic with symptoms consistent with irreversible pulpitis were screened for eligibility.

## Inclusion criteria:

[1] Age 18-65 years

[2] Mandibular first or second molars with symptomatic irreversible pulpitis

[3] Normal periapical radiographic appearance or periapical radiolucency ≤3 mm

[4] Positive response to cold testing with lingering pain

[5] Tooth restorable with adequate coronal structure

[6] American Society of Anesthesiologists (ASA) Physical Status I or II

## Exclusion criteria:

[1] Systemic diseases affecting pain perception (diabetes, neuropathies)

[2] Pregnancy or lactation

[3] Antibiotic or analgesic use within 7 days before treatment

[4] Previous endodontic treatment of the study tooth

[5] Presence of acute apical abscess with swelling

[6] Teeth with calcified canals or complex anatomy (S-shaped, severe curvatures)

[7] Periodontal disease with probing depths >5 mm

[8] History of chronic pain disorders or psychological conditions

## Randomisation and blinding:

A computer-generated randomisation sequence with block sizes of 4 and 6 was used to assign eligible patients to two different treatment groups randomly. The allocation concealment was ensured by sequentially numbered opaque and sealed envelopes that were prepared by an independent investigator not engaged in patient recruitment and treatment. Both operator and patient blinding could not be done because of the nature of the intervention. Nevertheless, the outcome assessor who was tasked with the responsibility of analysing the pain data and assessment was not made aware of the group assignment during the study.

## Clinical procedures:

The treatments were all carried out by one experienced endodontist who had over 10 years of clinical experience, so that the operator effect can be controlled to the minimum possible. Surgical practices were performed according to protocol under the isolation of a rubber dam.

## Anaesthesia protocol:

The amount of 2 per cent lidocaine mixed with 1:100000 epinephrine (1.8 mL) was used to perform the inferior alveolar nerve block. Any additional buccal infiltration (0.9 mL) was given when necessary. The process of the treatment was continued only after profound anaesthesia was confirmed by cold testing and patient feedback.

## Access cavity preparation:

Round diamond burs and Endo-Z burs were used to prepare standard access cavities under a lot of water irrigation. Any carious tissue was also abraded away and the pulp chamber was carefully debrided.

## Determination of working length:

Working length was determined with an electronic apex locator (Root ZX II, J. Morita Corp.) and ascertained radiographically at 0.5 mm below the radiographic apex.

## Instrumentation:

The manufacturer-recommended rotary files were used in a crown-down technique to ease the instrumentation of root canals with ProTaper Gold rotary files (Dentsply Sirona). End apical preparation was done in order to size F3 to the mesial and F2 to the distal canals. During instrumentation, apical patency was preserved with a size 10 K-file.

## Irrigation protocol:

The irrigation was done with 2.5 per cent sodium hypochlorite (20 mL per canal) by using side-vented needles with a 30-gauge opening placed 2 mm below the working length. End irrigation was done with 17% EDTA (3 mL per canal) in 1 minute and 2.5% sodium hypochlorite rinse. The canals were dried using sterile paper points.

## Group-specific procedures:

[1] Single-Visit Treatment (SVT): Canals were obturated during the same visit after instrumentation by warm vertical condensation technique using gutta-percha and AH Plus sealer (Dentsply Sirona). The access cavities were filled with provisional restorative material (Cavit G, 3M ESPE) and patients were to receive permanent restoration after two weeks.

[2] Multiple-visit treatment (MVT): After the instrumentation, inter-appointment dressing was done with calcium hydroxide paste (Calcipex II, Nippon Shika Yakuhin). After 7-10 days, Cavit G was used to seal the access cavities. Patients were returned to the clinic, where calcium hydroxide was eliminated through ultrasonically activated irrigation and then obturation was done as noted above.

## Instructions after surgery:

Standardised postoperative instructions were given to all patients with:

[1] No chewing on the treated side within 24 hours.

[2] Ibuprofen 400 mg to use as a rescue analgesic (do not exceed 3 times/day)

[3] Order to take analgesic intake.

[4] Contact information about the emergency.

Patients were highly advised not to use unprescribed analgesics and to report to the clinic in case of severe pain and swelling and in case of fever.

## Outcome assessment:

## Primary outcome:

The intensity of postoperative pain was determined on a 100-mm Visual Analogue Scale (VAS) with anchors of no pain (0 mm) and worst pain imaginable (100 mm). Pain scores were measured by the patients at 6, 12, 24, 48 and 72 hours and 7 days after completion of treatment.

## Secondary outcomes:

[1] Consumption of analgesics (quantity of tablets)

[2] incidence of flare-ups (defined as moderate to severe pain that necessitates an unscheduled appointment and /or swelling)

[3] Swelling (self-report and/or physical exam)

Categories of pain incidence: none (VAS 0), mild (VAS 1-30), moderate (VAS 31-54), severe (VAS 55 and above)

## Statistical analysis:

The analysis of data was conducted with the help of the SPSS software (version 26.0, IBM Corp.). The test of normal distribution was assessed by the Shapiro-Wilk test. Independent t-test and chi-square test were used to compare baseline characteristics on continuous and categorical variables, respectively. ANOVA was used to assess changes in the intensity of pain over time and between groups using repeated measures with the Greenhouse- Geisser correction. Non-normally distributed data were used in the Mann-Whitney U test. Compared categorical outcomes of groups, the chi-square or Fisher's exact test was used. The level of statistical significance taken was 0.05.

## Results:

Of 156 patients initially screened, 120 met the inclusion criteria and were randomised (57 per group). Six patients were lost to follow- up (3 per group), yielding 114 patients completing the study (57 per group). Reasons for dropout included failure to return pain diaries (n=4) and protocol violations (n=2). Baseline demographic and clinical characteristics were comparable between groups. Mean age, sex distribution, tooth location and preoperative pain intensity showed no significant differences (p>0.05). Pain intensity measurements across all time points are presented in Table 1 (see PDF). Both groups demonstrated similar pain patterns characterised by peak intensity at 6-12 hours post-treatment, followed by a gradual decline over subsequent days. However, no significant between-group difference (p=0.485), indicating comparable pain trajectories (Table 2 - see PDF). Pain incidence categorisation and secondary outcomes are summarised in Table 3 (see PDF) The proportion of patients experiencing no pain increased progressively in both groups over time. At 24 hours, 14.0% of SVT patients and 12.3% of MVT patients reported no pain, while 15.8% and 17.5%, respectively, reported severe pain. Flare-up rates were 6.7% (4/60) in the SVT group and 8.3% (5/60) in the MVT group, with no statistically significant difference (p=0.748). All flare-up cases were managed with additional analgesics and antibiotics where indicated, without requiring retreatment. Mean analgesic consumption was comparable between groups (SVT: 3.45 ± 2.12 tablets; MVT: 3.78 ± 2.34 tablets; p=0.412). The proportion of patients requiring at least one analgesic dose was similar (84.2% vs. 87.7%, p=0.588).

## Discussion:

This randomised clinical study showed that the use of single-visit and multiple-visit root canal treatment regimens has similar results in terms of postoperative pain in mandibular molars with symptomatic irreversible pulpitis. Treatment groups did not differ significantly on the intensity of pain, flare-up, the presence of swelling or analgesic intake, which underlines the clinical feasibility of single-visit endodontics. The patterns of pain that were similar in both groups, where the pain is the highest at 6-12 hours and then gradually decreases, are consistent with the inflammatory response pattern after endodontic procedures [15]. Subsequent decrease is an indicator of acute inflammation resolution and the tissue healing processes. The observation that there was no significant difference in the postoperative pain between the single and multiple-visit treatments is in support of other studies done in the past. Pak and White performed a meta-analysis showing the same incidence of short-term complications in visit protocols and the pooled odds ratio is near unity [16, 17]. Flare-up rates, which were experienced during this research (6.7% SVT, 8.3% MVT), are within the contemporary range [18]. The marginally increased (non-significant) flare-up rate in the multiple-visit group may reflect additional instrumentation trauma during calcium hydroxide removal at the second appointment. Contrary to traditional beliefs favoring multiple-visit superiority, a recent meta-analysis found no significant difference in success rates between single-visit and multiple-visit root canal therapy across 22 clinical trials. Single-visit protocols showed comparable long-term healing and only marginally higher early flare-up risk, supporting their clinical equivalence when chemomechanical debridement is thorough [19]. Nevertheless, modern thinking acknowledges that deep chemomechanical debridging attains significant bacterial elimination irrespective of further medication and immediate obturation averts recontamination due to the temporary restoration failures [20]. The similar levels of analgesic use among the groups also give further support to the same experiences of pain. The mean analgesics used by patients in both groups were around 3-4 analgesic pills during the period of observation and it reflected moderate pain which was effectively controlled using over the counter painkiller [21]. A similar result might be explained by a number of factors. Modern rotary instrumentation can be used to achieve efficient, standardised canal preparation to reduce debris extrusion and minimise the time spent in the procedure as opposed to the traditional method [22]. Moreover, better irrigation guidelines with activation techniques increase the efficacy of antimicrobials, lessening the remaining bacterial load, which may normally trigger the occurrence of postoperative complications [23]. The clinically relevant and homogeneous study population was the selection of mandibular molars with symptomatic irreversible pulpitis. Teeth containing vital pulp tissue and having minimal periapical involvement might make ideal candidates for single-visit treatment since bacterial contamination is limited only to the coronal pulp space [24]. On the contrary, teeth with already developed apical periodontitis can be treated with inter-appointment medication to overcome more widespread microbial colonisation, but the argument in favour of this statement is still disputable [25]. The consideration of patients will lead to the implementation of single- visit guidelines where it is clinically relevant. Fewer appointments mean less inconvenience to the patients, less time out of work or school and less healthcare expenditure due to multiple visits [26]. Additionally, the eradication of inter-appointment periods eliminates the chances of temporary loss of restoration and later recontamination of the canal. The clinical implications of the presented findings relate to the treatment planning and communication with the patient. Practitioners can give single- visit treatment with confidence to the right patients and can give evidence-based assurance to the patients on the outcome after the operation. Clinical judgment, however, is still necessary and the multiple-visit protocols can be the choice in situations of complex cases, medically compromised patients, or when time constraints do not allow the comprehensive treatment of a patient in a single appointment [27]. There are a number of constraints of this work that should be mentioned. The single-site design and rather brief follow-up restrict the applicability and cannot evaluate extended curing results. Also, the results emphasized in a particular type of tooth and diagnosis may not be applicable in other clinical situations. The skill of an operator can also be an issue since the standardised protocol was introduced by a qualified endodontist. The next round of multicenter studies that focus on a wide range of clinical manifestations and follow-up would complement the evidence base [28].

## Conclusion:

In the constraints of this randomised clinical trial, cross-sectional and cross-sectional root canal therapies showed the same post- operative pain, exacerbation frequencies and analgesic demands in the mandibular molars with symptomatic irreversible pulpitis. The two protocols were both clinically safe and they gave a similar patient experience with no huge disparities in postoperative complications. Thus, it is possible to discuss single-visit root canal treatment as a reliable and time-saving option and the selection of the protocol must rely on the clinical judgment and preferences of the patient.
